# MicroRNA‐502‐3p, a novel regulator of GABA function in Alzheimer’s disease

**DOI:** 10.1002/alz.087754

**Published:** 2025-01-09

**Authors:** Bhupender Sharma, Melissa Torres, Sheryl Rodriguez, Subodh Kumar

**Affiliations:** ^1^ Texas Tech University Health Sciences Center El Paso, El Paso, TX USA

## Abstract

**Background:**

In various neurological disorders, including Alzheimer’s disease (AD) and AD‐related dementia, there is a notable reduction in gamma‐aminobutyric acid (GABA)ergic neurons, which represent the most abundant inhibitory neurons in the human brain. This study explores molecular association between miR‐502‐3p and the function of GABAergic neurons in AD.

**Method:**

The investigation commenced by examining the status of GABA receptor proteins and miR‐502‐3p in postmortem AD brains. Subsequent in‐vitro experiments were conducted using the mouse hippocampal neuronal (HT22) cells, transfected with miR‐502‐3p agomiRs and antagomiRs. In‐silico analysis, performed to select miR‐502‐3p potential targets. Thereafter targeting of the GABRα1 gene by miR‐502‐3p were confirmed through a different technique, including QRT‐PCR, western blotting and ELISA, cell viability, miRNA in‐situ hybridization, immunoblotting, and immunostaining techniques were used to confirm the effect of miR‐502‐3p overexpression on the GABRα1 level and GABRα1 proteins.

**Result:**

Reduced levels of GABA receptor subunits correlating with Braak stages were identified in AD postmortem brain samples compared to control. We found upregulated miR‐502‐3p expression in AD postmortem brain samples, correlating with Braak stages. In‐silico analysis, revealed multiple binding sites of miR‐502‐3p at GABRα1 mRNA. The targeting of the GABRα1 gene by miR‐502‐3p were confirmed through luciferase assay, QRT‐PCR, western blotting and ELISA for GABRα1. Elevated miR‐502‐3p levels showed toxic effects on HT22 cells and reduced cell viability. Furthermore, miRNA in‐situ hybridization, immunoblotting, immunostaining and whole cell patch‐clamp analysis confirmed that miR‐502‐3p overexpression (miR‐502‐3p agomiRs) suppressed GABRα1 mRNA and protein levels. Conversely, suppression of miR‐502‐3p (miR‐502‐3p antagomiRs) resulted in the restoration of GABRα1 levels, an increase in GABA current, and an overall enhancement of GABA function. Additionally, enhanced levels of proteins associated with AD were found with miR‐502‐3p overexpression and reduced with miR‐502‐3p suppression.

**Conclusion:**

The study uncovered an inverse correlation between miR‐502‐3p levels and GABA subunit proteins. Notably, inhibition of miR‐502‐3p demonstrated a protective effect on GABA function. Collectively, these findings suggest that miR‐502‐3p could serve as a promising therapeutic target for enhancing GABAergic neuron function in neurological disorders, including AD and AD‐related dementia.

